# The "Map of Life."

**Published:** 1899-10-28

**Authors:** 


### The "Map of Life."

All who are familar with the earlier works of Mr. Lecky will be prepared to welcome the chapters on "Conduct and Character," which, under the somewhat disguising title of "The Map of Life," he has just issued from the press. To those earlier works the present generation has been indebted for a lucid exposition of the manner in which the public estimate of conduct becomes insensibly modified by under-currents of opinion which it is often difficult to trace to the sources in which they originate, or even to observe and identify until after they have produced great and far-reaching changes—changes which, for the most part, cannot be attributed either to the acquisition of fresh knowledge on the subjects affected by them, or to the enforcement of pre-existing knowledge by new and better arguments. By some silent and unfelt operation "the old order changeth, giving place unto the new"; and opinions which once prevailed extensively, and which controlled vast fields of human action, somehow fall away from the minds of the descendants of those by whom they were strongly held and strenuously enforced. No one can point to the beginning of the change, no one can deny or overlook its completion; and, in these respects, there is an almost precise parallelism between organic and inorganic nature, between the world of physics and the world of thought:

> "Who ever saw the earliest rose
> First open her sweet breast;
> Or, when the summer sun goes down,
> The first bright star in evening's crown
> Light up its gleaming crest?"

Madame de Maintenon was, perhaps, scarcely accurate when she said that the difference between right and wrong was mainly a matter of geography; but her error was in not perceiving that the essential difference was one of chronology, rendered apparently geographical by the fact that certain localities were in advance of others in all that related to civilisation and enlightenment. But, even under the most advanced civilisation, there can be no finality, and our countrymen are not only daily and unconsciously escaping from errors to which their ancestors were prone, but may also very possibly be falling into others from which their ancestors were exempt. We have no assurance that change must of necessity be improvement; and it is very salutary that such a book as that of Mr. Lecky should sometimes bring us to our bearings, and compel us to consider our moral status by comparison with that of other countries or of earlier generations.

There is perhaps no profession or calling in which character and conduct are of so much importance as in the medical, and there is certainly none in which even the first principles of ethics are at present in so semi-fluid or plastic a condition. This is perhaps partly due to the circumstance that lessons in the principles which should govern professional conduct have no place in the modern scheme of medical education, so that every young practitioner is almost compelled to be a law unto himself, and to deal with any difficult position in which he may be placed by the aid of such light as he may obtain from his own conception of general moral requirements, or under the influence of any tendencies which may be the outcome of his own disposition, selfish or altruistic, as the case may be. One man will be chiefly actuated by what he conceives to be his duty to himself, another by what he conceives to be his duty to his neighbour. Another unsettling influence, if we may so describe it, arises from the temptations which are placed in the path of medicine by the "non-morality," to use Professor Huxley's convenient word, of modern plutocracy. The "fashionable" doctor is treated by the plutocracy, and especially by those members of it who aspire to be conspicuous in "society," with as little consideration as if the ladies, who are usually most actively concerned, had no more sense of moral responsibility than monkeys. The effect is to place before young men, on their entrance into the profession, conspicuous examples of pecuniary success which is independent of knowledge and skill, of which the fair patronesses cannot be judges, and dependent only upon *savoir-faire*, or, it may be, only upon something called a "system," or a "treatment," which has "caught on" with "smart" people. The money earned in this way is the more tempting to certain dispositions, inasmuch as the patients have usually but little the matter with them, and the fees are obtained without any burden of responsibility. In provincial districts, again, the moral sense of the practitioner is apt to be sorely tried by the endeavours of certain classes of residents to over-reach or get the better of him, to become members of benefit societies when they are well able to pay, or to cut down his remuneration for the performance of public duties to the lowest possible point. It is much to be wished that some of the real leaders of profe sional opinion would give their minds to the classes of questions hence arising; and would devote, to the instruction of students in the principles of medical conduct, some small portion of the time which is now occupied in teaching them other and not necessarily more useful things.

